# Auricular Plaster Therapy for Comorbid Insomnia: A Systematic Review and Meta-Analysis of Randomized Controlled Trials

**DOI:** 10.1155/2019/7120169

**Published:** 2019-01-15

**Authors:** Huimin Zhao, Dan Li, Ying Yang, Yueting Liu, Jie Li, Jing Mao

**Affiliations:** ^1^School of Nursing, Tongji Medical College, Huazhong University of Science and Technology, Hangkong Road 13, Qiaokou District, Wuhan 430030, China; ^2^Department of Pathology, Taihe Hospital, Hubei University of Medicine, No 32 South People's Road, Shiyan 442000, China

## Abstract

**Background:**

Although the effectiveness of auricular plaster therapy (APT) on primary insomnia has been systematically reviewed, no systematic review of studies has focused on the effect on comorbid insomnia.

**Objective:**

To evaluate the efficacy and safety of APT for comorbid insomnia.

**Methods:**

Fifteen databases were searched from inception to July 2018. Randomized controlled trials (RCTs) of APT as an exclusive intervention for comorbid insomnia against Western medications, sham APT or no treatment were identified.

**Results:**

Fourteen studies involving 928 participants were identified. The pooled outcomes revealed that APT was superior to control conditions for the global score on PSQI (SMD = -1.13, and 95% CI = -1.48—-0.78) and the effective rate (RR = 1.24, 95% CI = 1.13—1.36, NNT = 5, and 95% CI =4—7). Furthermore, the results of subgroup analyses were similar to the pooled results. Additionally, the pooled results were verified to be stable by sensitivity analyses. Regarding safety, no significant difference was identified between APT and Western medications.

**Conclusions:**

APT appears to be an effective and safe treatment for comorbid insomnia. However, the benefits of APT for comorbid insomnia could not be ascertained due to the paucity of the quantity and quality of the included studies. Large-scale studies using proper methodology are needed to yield a firm conclusion.

## 1. Introduction

Insomnia is one of the most major public health problems, which is characterized by difficulty initiating or maintaining sleep, and early morning waking with significant impairment in daytime functioning [1]. Historically, insomnia has been classified as primary and secondary insomnia [2, 3]. Primary insomnia is defined as insomnia without co-morbidity [4]. Secondary insomnia is the term used when insomnia is associated with another disorder [2, 3]. However, the term “secondary insomnia” often leads to underdiagnosis and undertreatment. Therefore, the National Institute of Health State of the Science Conference proposed that “secondary insomnia” should be renamed “comorbid insomnia” [4]. Comorbid insomnia is subdivided into insomnia associated with another medical or psychiatric disorder [5]. Accordingly, medical conditions including diabetes, coronary heart disease, chronic obstructive pulmonary disease, arthritis, fibromyalgia, and other conditions, and psychiatric disorders such as bipolar, depressive, anxiety, and other disorders are considered risk factors for comorbid insomnia [5].

Comorbid insomnia is even more common than primary insomnia [6]. Studies have found that the prevalence of insomnia in patients with medical disorders varies extensively from 22% to 81% [7–12]. The prevalence of insomnia also changes from 24% to 93% [13–18] in patients with psychiatric disorders. The economic, health, and functional implications of comorbid insomnia are substantial, such as delay in recovery [19], role impairment [20], and increased healthcare costs [21]. Given the high prevalence and detrimental effects of comorbid insomnia, it is critical to identify effective, acceptable, and affordable treatment strategies for patients.

Common treatments for insomnia include pharmacological therapy, psychological therapy, and complementary and alternative medicine (CAM). Pharmacological therapy is one of the most frequent interventions used in clinical practice for relieving insomnia [4]. The main form of psychological therapy is cognitive behavioural therapy (CBT) that has been proven to be an effective treatment [22]. However, each therapy has specific limitations. Pharmacotherapy such as benzodiazepines and nonbenzodiazepine hypnotics is associated with potential adverse effects, drug interactions, and substance abuse [22–25]. The accessibility and availability of CBT are limited by the number of trained practitioners and the cost of time [26, 27]. Consequently, CAM has become an option for patients with insomnia. A national survey in 2002 found that greater than 1.6 million American adults used CAM to treat insomnia or trouble sleeping during the past year [28].

Auricular therapy is one treatment modality of CAM, which refers to the stimulation at specific acupoints on the outer ear to facilitate recovery of health [29]. The practice originates from ancient China, with a history of more than two thousand years [30].

APT is considered as a form of auricular therapy in which small, round, hard smooth objects with appropriate size are attached to the auricular points [31]. Although the mechanisms of APT to treat comorbid insomnia have not yet been elucidated, there are many studies suggesting that APT may improve insomnia by acting on the nervous systems and modulating the activities of neurotransmitters [32]. APT is often practiced by health care workers, patients themselves, or family members perhaps due to its non-invasive nature, safety and convenience. Objects such as Semen Vaccariae (SV) and magnetic pellets (MP) are typically used in auricular attachment.

To date, there is growing evidence that APT can be applied to treat a variety of disorders. In 1976, a group of researchers from Jiangsu New Medical College successfully used APT to treat flat warts [33]. Subsequently, the research on APT has been extended to manage pain [34], constipation [35, 36], hypertension [37, 38], diabetes [39], myopia [40, 41], and other conditions. Similarly, previous studies indicated a significant improvement in insomnia [42, 43].

The first systematic review was initiated in 2007, performed by Chen and colleagues to demonstrate the favourable effects of APT on primary insomnia [44]. Since its publication, several new studies have been published. A recent meta-analysis published in 2015 examined the benefits of APT for primary insomnia and included fifteen studies [45]. In brief, these systematic reviews or meta-analyses demonstrated that APT appeared to be efficacious for improving sleep quality for patients with primary insomnia [44–48]. However, one limitation common to these studies is poor methodological quality. In recent years, although the number of published studies regarding the use of APT in patients with comorbid insomnia has steadily increased, the efficacy and safety of APT for comorbid insomnia remain uncertain.

To our knowledge, there has been no systematic review or meta-analysis summarizing the therapeutic role of APT for comorbid insomnia to date. Given the significant health risk of comorbid insomnia and increasing interest in APT, it is essential to accumulate current evidence on the effects of APT on comorbid insomnia. To that end, the objective of the present study was to summarize the efficacy and safety of APT for the treatment of comorbid insomnia.

## 2. Methods

### 2.1. Search Strategy

This study was planned, performed and reported in compliance with the Preferred Reporting Items for Systematic Reviews and Meta-Analyses (PRISMA) guidelines [49]. We systematically searched MEDLINE, EMBASE, Pubmed, PsycINFO, Cochrane library, Cochrane Central Register of Controlled Trials, ProQuest Dissertations and Theses, Cumulative Index to Nursing and Allied Health Literature, and Allied and Complementary Medicine from inception to July 26, 2018 using the grouped terms (insomnia*∗* OR sleep*∗* OR sleepless*∗* OR wakeful*∗*) AND (auricular*∗* OR acupress*∗* OR acupuncture*∗* OR acupoint*∗*). The search also included Chinese National Knowledge Infrastructure, Chinese Biomedical Database, Chinese Scientific Journal Database, Wanfang Data, China Doctor Dissertations Full-text Database, and China Master Theses Full-text Database using the Chinese terms (*失眠* OR 不寐 OR 不得眠 OR 不得卧 OR 目不瞑 OR* 睡眠障碍*) AND (*耳穴贴压* OR* 耳穴按压* OR* 耳穴刺激* OR* 耳穴埋豆* OR* 耳穴压豆* OR* 耳穴压丸* OR* 耳穴埋籽* OR* 磁珠贴压* OR 王不留行*籽贴压*). In addition, the reference lists of eligible studies were searched by hand.

### 2.2. Inclusion and Exclusion Criteria

Studies were eligible for inclusion if they fulfilled the following criteria: (1) RCTs focused on the comparison of APT as monotherapy with Western medications, sham APT, placebo or no treatment for comorbid insomnia regardless of two-arm or multi-arm studies; (2) comorbid insomnia is defined as insomnia associated with additional medical or psychiatric disorders [5]. Thus, insomnia patients with one or more medical or psychiatric disorders were included. No restrictions regarding age, gender, nationality, or ethnic background were applied; and (3) the primary outcome was measured by the Pittsburgh Sleep Quality Index (PSQI). The PSQI is a 19-item self-rated questionnaire that consists of seven domains. Each domain score varies from 0 to 3. Seven domains are added to generate a global PSQI score, ranging from 0 to 21. Higher scores indicate worse quality of sleep [50]. The secondary outcome was measured by effective rate. And/or adverse events were reported as the safety outcome. Studies were excluded if they (1) were duplicate records (i.e., the publication of an article overlapping substantially with an article published elsewhere) [51]; (2) reported inadequate data (i.e., deficiency of statistical information regarding PSQI or effective rate); (3) only compared different forms of APT; or (4) assessed the effectiveness of the combination of APT and other therapeutic methods for comorbid insomnia.

### 2.3. Study Selection

First, titles of all articles were independently reviewed by two authors (H.M.Z. and Y.Y.) to eliminate irrelevant publications. Then, the abstracts of possibly relevant studies were reviewed. Finally, the full-text articles of all candidate studies were read. Discrepancies at each step were resolved through discussion and consultation with another author (J.L.).

### 2.4. Data Extraction

Data regarding the following aspects were extracted from the selected full-text articles by two authors (H.M.Z. and Y.T.L.): study characteristics (authors, year of publication, and sample size), patient characteristics (age, gender, and duration of insomnia), study methods (details of intervention and control), intervention protocols (timing, frequency and duration of treatment), and outcome measures (PSQI, effective rate, and adverse events). A third author (J.L.) validated the final dataset. We attempted to contact authors by e-mail to obtain missing information.

### 2.5. Quality Assessment

Cochrane's risk of bias assessment tool was used to evaluate the quality independently by two authors (H.M.Z. and Y.Y.). Cochrane's risk of bias assessment tool included the following seven components: random sequence generation, allocation concealment, blinding of personnel and participants, blinding of outcome measurement, incomplete outcome data, selective reporting, and other sources of bias. Each component was categorized as low, high, or unclear risk of bias [52].

The Jadad scale [53] was also used to assess the study quality according to the description of randomization, double blinding, and withdrawals and dropouts, resulting in a score of up to 5 points. Study quality was rated as low (≤2) or high (≥3).

### 2.6. Statistical Analysis

Statistical analyses were conducted with Review Manager (version 5.3) and STATA (version 14.0). Risk ratio (RR) was summarized as the effect size for dichotomous outcomes, and standardized mean difference (SMD) was calculated for continuous outcomes. Both values were reported with 95% confidence interval (CI). When RR was significant, we calculated the number needed to treat (NNT) with 95% CI. Heterogeneity across studies was investigated using Chi-square-based Q test and* I*^2^ statistics. Considering generally low statistical power of heterogeneity tests, statistical significance was set at a more liberal* P *< 0.1. The extent of heterogeneity among studies was quantified using* I*^2^ value and classified as low (0–40%), moderate (30–60%), substantial (50–90%), and considerable (75–100%) [54]. Subgroup analyses were performed to explore the sources of heterogeneity by different control methods and the type of APT. The sources of heterogeneity were also investigated with meta-regression, if at least ten studies were included in the analysis [54]. To verify the stability of the pooled results, sensitivity analyses were undertaken by interchanging statistical models and individually removing the included studies. The random-effect model was chosen to pool the data due to different comorbidities, diagnostic criteria used for insomnia and treatment regimens among the included studies. Publication bias was examined qualitatively by constructing funnel plots and objectively using the Egger's test (*P* < 0.10 indicates the presence of publication bias) if at least ten studies were available in the analysis [54, 55]. Two-sided* P *< 0.05 denoted statistical significance.

### 2.7. Quality of Evidence

Two authors (HMZ and DL) independently used the Grading of Recommendations Assessment, Development and Evaluation (GRADE) approach to rate the quality of evidence for each outcome as high, moderate, low, or very low [54]. RCTs begin as high quality evidence but can be downgraded based on the following five factors: (1) risk of bias; (2) unexplained heterogeneity or inconsistency of results; (3) indirectness of evidence; (4) imprecision of results; and (5) publication bias. Differences in quality of evidence were resolved through discussion and consultation with a third author (JL).

## 3. Results

### 3.1. Study Selection

**Figure 1:**
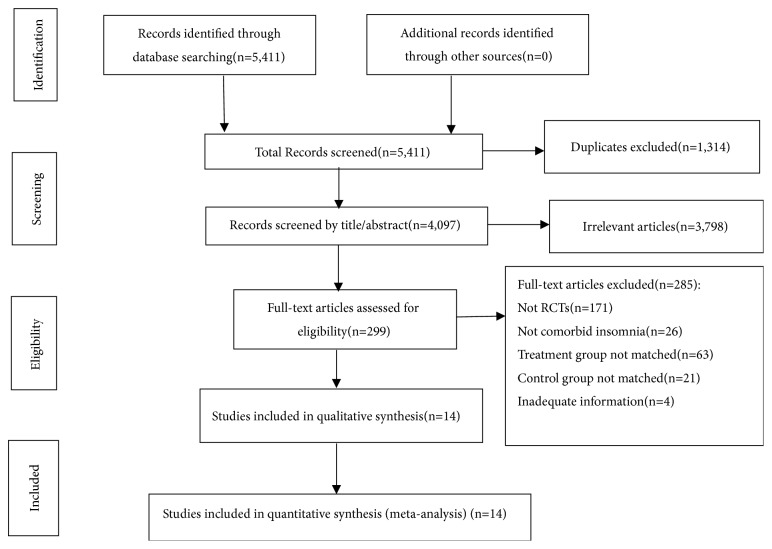
Literature search flow diagram.

The flow diagram of the study identification, screening, eligibility, and inclusion was presented in Figure 1. The initial search identified 5,411 citations using electronic databases and manual searching, and 1,314 duplicates were excluded. Then, 3,798 articles were eliminated for reasons of irrelevance after screening the titles and abstracts. The full texts of the remaining 299 articles were retrieved for detailed review, and 285 were excluded for several reasons. In total, fourteen studies [56–69] met the eligibility criteria for inclusion.

### 3.2. Study Characteristics

The demographic, clinical, and therapeutic characteristics of included studies were presented in Tables 1 and 2. The fourteen studies included 928 participants ranging from 18 to 95 years. All of the studies were conducted between 2012 and 2018. Only one was a multi-arm study, whereas the remaining were two-arm studies. The sample sizes of the studies ranged from 40 to 155, and patients had a variety of comorbidities, such as hypertension [68], diabetes [60, 61], post-stroke [62, 63], cerebral stroke [65], acute cerebral infarction [59], hepatocirrhosis [56], maintenance hemodialysis [57, 67], chronic obstructive pulmonary disease [64], and hip fracture [66, 69].

With respect to the criteria used for the diagnosis of insomnia, Criteria of Diagnosis and Therapeutic Effect of Diseases and Syndromes in TCM was used in four of the fourteen studies [56, 57, 60, 68], Chinese Classification of Mental Disorder was adopted in three studies [61, 63, 67], Diagnostic and Statistical Manual of Mental Disorders was employed in one study [58], PSQI was employed in two studies [61, 67], Self-rating Scale on Sleep was used in one study [62], and textbooks, including* 《*Neurology》and《Internal Medicine of Traditional Chinese Medicine》, were used in four studies [59, 64, 65, 67]. Two studies did not report the diagnostic methods employed [66, 69].

Comparison conditions differed in studies. The fourteen studies comprised twelve comparisons [57–63, 65–69] with 778 patients of APT against Western medications (such as estazolam and diazepam) and two comparisons [56, 64] with 150 patients of APT against sham APT (such as using adhesive tape alone on auricular points) and no treatment (such as not receiving any treatment). The types of APT also varied among studies. In our study, the most commonly used form was SV [57–60, 62–69] in twelve studies, and the remaining two studies chose MP [56, 61]. In addition, acupressure technique differed across studies. The number of main acupoints varied from one to seven, with an average of 4.3 acupoints. The most frequently used acupoints were Shenmen, Subcortex, Heart, and Sympathetic. Similarly, regarding treatment protocols, timing, frequency, and duration of pressing exhibited some variation among studies. APT was given over a range from half to five minutes each time. The treatment was delivered three to five times per day for six to sixty days.

End points were measured inconsistently across studies. The outcome included global score on PSQI in one study [60], effective rate in five studies [56, 59, 62, 66, 69], and both in eight studies [57, 58, 61, 63–65, 67, 68]. Additionally, follow-up data were not included in analysis due to absence of reporting information about the long-term effects of APT on insomnia in all studies.

### 3.3. Quality Assessment

#### 3.3.1. Assessment Using the Cochrane Risk of Bias

**Figure 2:**
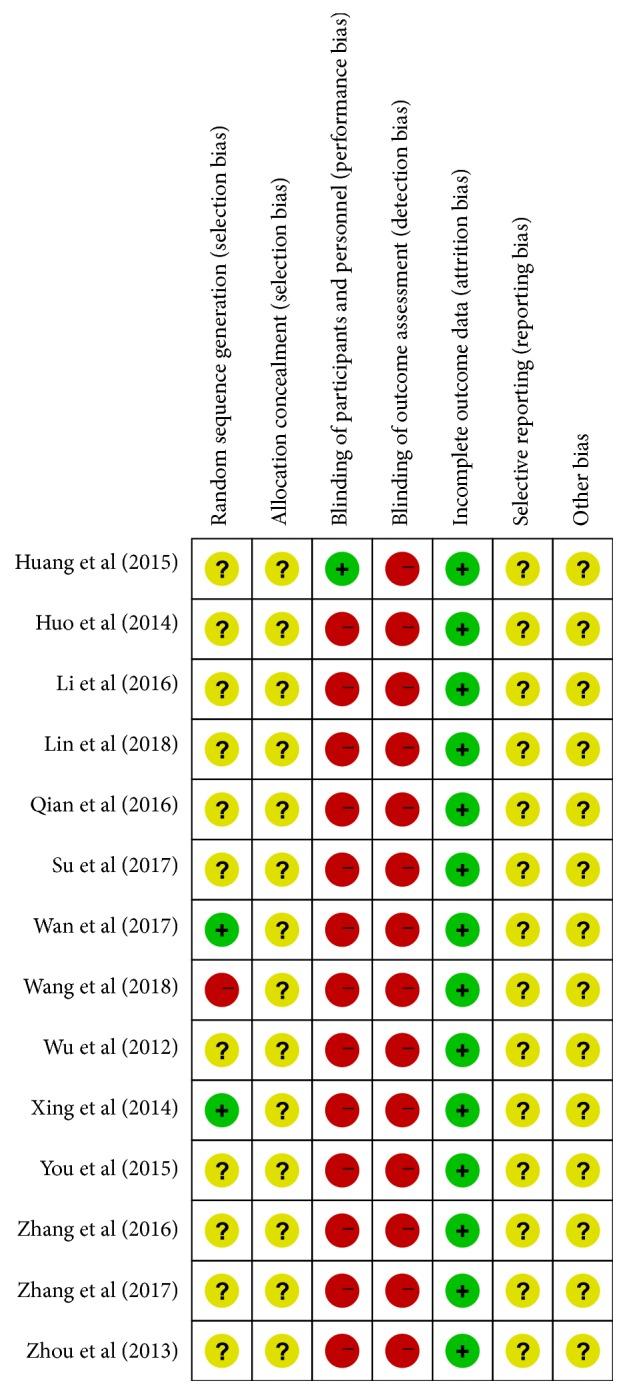
Risk of bias summary.

Overall, the fourteen studies were determined to have significant risk of bias (Figure 2). Most of the studies lacked sufficient information to assess the risk of bias. All studies were randomized, but only two studies [61, 63] provided the details of sequence generation by random number table. None of the studies explicitly stated that treatment allocation was concealed. With the exception of one study [56] that adopted a sham control to blind the personnel and participants, masking of personnel and/or participants was not performed in the remaining studies. This limitation may be due to the fact that performing blinding methods might be difficult for different forms of APT and control intervention. However, all studies were free of incomplete outcome data. For selective outcome reporting, all studies were rated as an unclear risk because trial registration or a protocol was unavailable.

#### 3.3.2. Assessment Using the Jadad Scale

In general, among the fourteen studies, the mean Jadad score was 1.5 (Table 3), indicating that the study quality was low. Two studies had a Jadad score of 3 [56, 61], three scored 2 [63, 65, 68], and nine scored 1 [57–60, 62, 64, 66, 67, 69].

### 3.4. Efficacy and Safety Outcomes

#### 3.4.1. Primary Outcome-Global Score on PSQI

**Figure 3:**
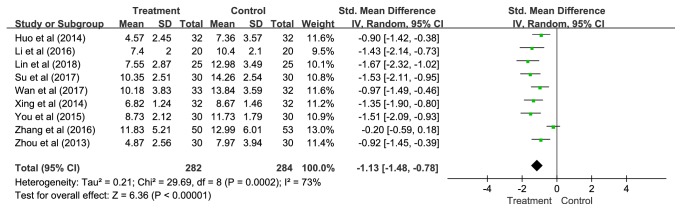
The pooled results of global score on PSQI.

*(1) Overall. *Nine studies presenting data of 566 participants were included in this pooled analysis. The overall results showed that APT was significantly more effective than control groups (SMD = -1.13, 95% CI = -1.48—-0.78, and* P *< 0.00001), but statistical heterogeneity was noted across studies (*P *= 0.0002;* I*^2^= 73%) (Figure 3).

To explore potential sources of this heterogeneity, subgroup analyses were performed according to different control methods and the type of APT.

**Figure 4:**
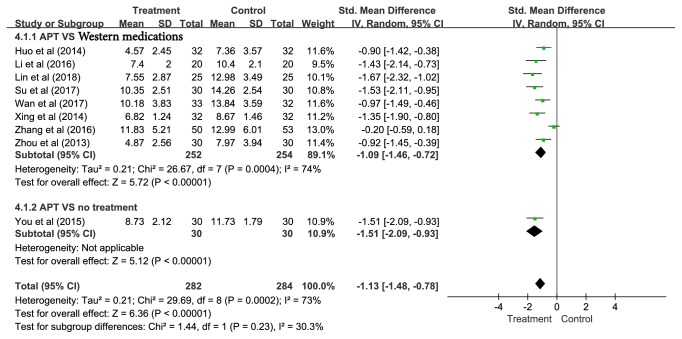
Subgroup analyses of global score on PSQI according to different control methods.

*(2) Different Control Methods. *Control methods included Western medications (i.e., estazolam and diazepam) [57, 58, 60, 61, 63, 65, 67, 68] and no treatment (i.e., not receiving any treatment) [64]. Eight studies revealed that APT achieved better outcomes compared with the Western medications (SMD = -1.09, 95% CI = -1.46—-0.72, and* P *< 0.00001), and one study showed that APT had better efficacy compared with no treatment (SMD = -1.51, 95% CI = -2.09—-0.93, and* P *< 0.00001) (Figure 4).

**Figure 5:**
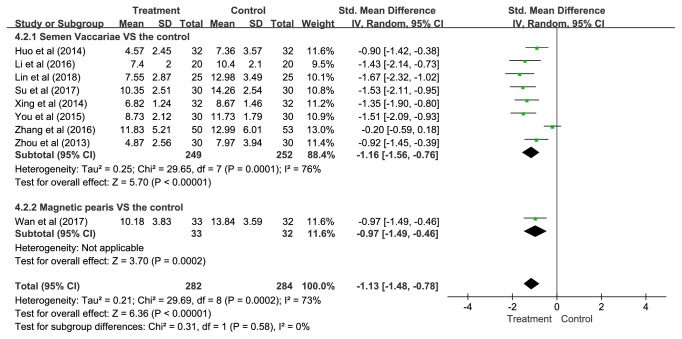
Subgroup analyses of global score on PSQI according to the type of APT.

*(3) The Type of APT. *Eight studies compared SV with control conditions and one study compared MP with a control group. In summary, regardless of SV or MP, APT was significantly superior to controls (SV: SMD = -1.16, 95% CI = -1.56—-0.76, and* P *< 0.00001; MP: SMD = -0.97, 95% CI = -1.49—-0.46, and* P *= 0.0002) (Figure 5).

#### 3.4.2. Secondary Outcome-Effective Rate

**Figure 6:**
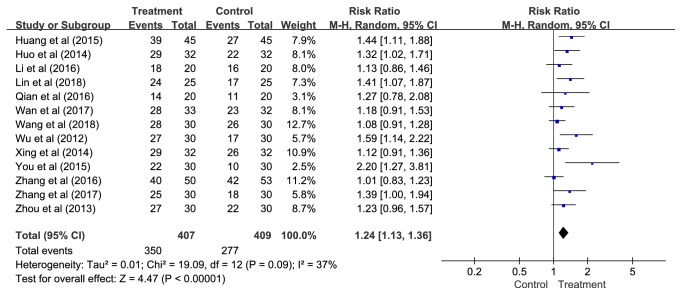
The pooled results of effective rate.

*(1) Overall. *Thirteen studies containing results for 816 patients were identified in this pooled analysis. The pooled estimates suggested that APT had a much better therapeutic effect compared with control conditions (RR = 1.24, 95% CI = 1.13—1.36,* P *< 0.00001, NNT = 5, and 95% CI =4—7) without statistical heterogeneity, but low to moderate heterogeneity was observed (*P *= 0.09;* I*^2^= 37%) (Figure 6).

To account for the observed heterogeneity, subgroup analyses and meta-regression were implemented based on different control methods and the type of APT.

**Figure 7:**
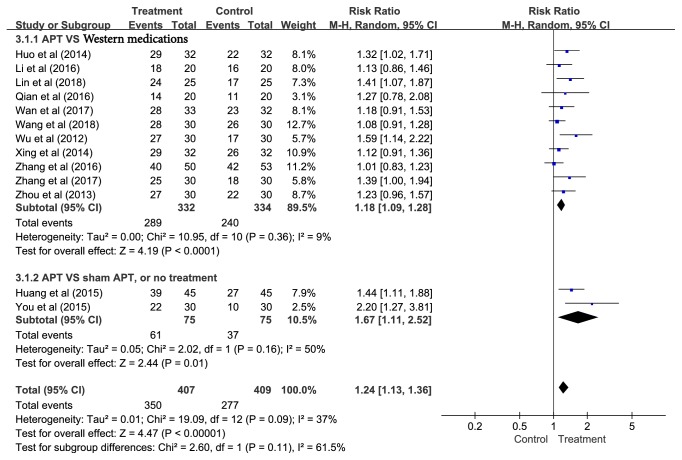
Subgroup analyses of effective rate according to different control methods.

*(2) Different Control Methods. *Control methods included Western medications (i.e., estazolam and diazepam) [57–59, 61–63, 65–69], sham APT (i.e., using adhesive tape alone on auricular points) [56], and no treatment (i.e., not receiving any treatment) [64]. Subgroup analyses demonstrated that APT had better effective rate compared with the Western medications (RR = 1.18, 95% CI = 1.09—1.28,* P *< 0.0001, NNT = 6, and 95% CI = 4—10), sham APT, or no treatment (RR = 1.67, 95% CI = 1.11—2.52,* P *= 0.01, NNT = 3, and 95% CI = 2—5) (Figure 7), although the* P-*value was not significant in meta-regression (*P *= 0.073).

**Figure 8:**
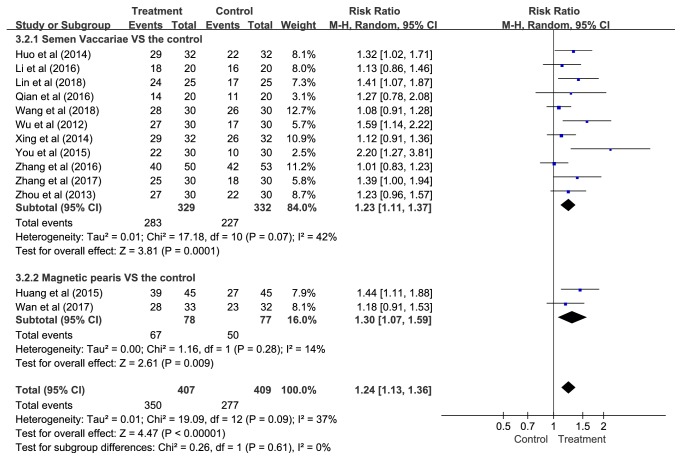
Subgroup analyses of effective rate according to the type of APT.

*(3) The Type of APT. *Eleven studies compared SV with control conditions and two studies compared MP with control groups. These results suggested that APT was statistically favoured over controls (SV: RR = 1.23, 95% CI = 1.11—1.37,* P *= 0.0001, NNT = 5, and 95% CI = 4—8; MP: RR = 1.30, 95% CI = 1.07—1.59,* P *= 0.009, NNT = 4, and 95% CI = 2—12) (Figure 8), although the* P-*value was not significant in meta-regression (*P *= 0.555).

#### 3.4.3. Safety Outcome-Adverse Events

**Figure 9:**
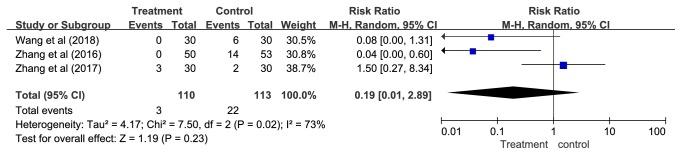
The pooled results of adverse events.

Three studies using Western medications as the control reported adverse events [65, 66, 69]. In one study [69], no adverse events were observed in the APT group and six cases of adverse events were reported in the control group (one case of fatigue, two cases of dry mouth, and three cases of mild dizziness). Zhang [65] reported no adverse events related to APT and 14 cases of adverse events in the control group (12 cases of mild dizziness and two cases of severe dizziness). Among them, two patients withdrew from the study due to severe dizziness. The other study [66] reported three cases of local pain in the treatment group and two cases of vomiting in the control group. The results of our meta-analysis revealed no significant difference in adverse effects between APT and Western medications (RR = 0.19, 95% CI = 0.01—2.89,* P* = 0.23, and* I*^2^= 73%) (Figure 9).

### 3.5. Sensitivity Analyses

First, the sensitivity analysis was performed by interchanging the random-effect and fixed-effect model, and the pooled outcomes of global score on PSQI (random-effect: SMD = -1.13, 95% CI = -1.48—-0.78, and* P *< 0.00001; fixed-effect: SMD = -1.02, 95% CI = -1.20—-0.84, and* P *< 0.00001) (Table 4(a)) and effective rate (random-effect: RR = 1.24, 95% CI = 1.13—1.36, and* P *< 0.00001; fixed-effect: RR = 1.27, 95% CI = 1.18—1.37, and* P *< 0.00001) (Table 5(a)) were considered robust.

Second, by omitting one study at a time and calculating a pooled result for the remaining studies, the estimates of global score on PSQI (Table 4(b)) and effective rate (Table 5(b)) remained relatively similar, suggesting minimal effects from individual studies.

### 3.6. Publication Bias

In terms of primary outcome, there were not sufficient studies to test publication bias.

**Figure 10:**
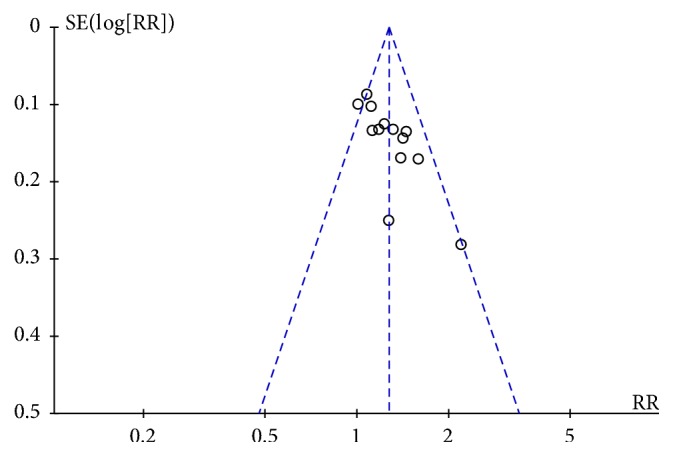
Funnel plot for evaluation of publication bias.

With regard to secondary outcome, the funnel plot showed slight asymmetry through visual inspection (Figure 10). We further quantitatively assessed the publication bias using the Egger's test. The result suggested no statistically significant publication bias in the current meta-analysis (*P* = 0.294).

### 3.7. Quality of Evidence

The quality of evidence for global score on PSQI was rated as low because of risk of bias and inconsistency; the quality of evidence for effective rate was graded as moderate because of risk of bias; and the quality of evidence was very low for adverse events because of risk of bias, inconsistency, and imprecision (Table 6).

## 4. Discussion

### 4.1. Summary of Main Evidence

This systematic review and meta-analysis, which comprised fourteen RCTs that included 928 participants, aimed to examine the effects and safety of APT in patients with comorbid insomnia. Of the included RCTs, eight reported data on effective rate and global PSQI score, five provided data on effective rate, and only one presented data on global PSQI score. Overall, our pooled results demonstrated statistically significant differences between APT and control conditions for both global PSQI score and effective rate. Moreover, subgroup analyses based on different control methods and the type of APT were in favour of APT. According to our results, APT resulted in a meaningful improvement in sleep quality relative to Western medications, sham APT or no treatment. Furthermore, regardless of SV or MP, APT provided a significant beneficial effect on sleep efficacy relative to control conditions for outcome measures. Sensitivity analyses further confirmed the robustness of the pooled results. More specifically, the pooled results did not appreciably change when comparing the random-effect and fixed-effect estimates. In addition, the summary effect size estimates did not differ substantially after individually excluding one study of eligible studies, which indicated that the overall outcomes were not dramatically influenced by a single study. With regard to safety outcome, the results indicated no statistically significant difference between APT and Western medications. We found that the majority of the RCTs included in this study were deemed to be of low quality and the quality of evidence for each outcome was not high. Hence, it is premature to conclude that APT is indeed an effective and safe strategy to treat comorbid insomnia.

### 4.2. Mechanisms of APT

The mechanisms of APT for insomnia include effects on the nervous systems and modulations of the activities of neurotransmitters [32]. First, insomnia is related to sympathetic hyperactivity [70]. APT may increase cardiac parasympathetic activity and decrease the sympathetic activity and thus improves insomnia [71]. Besides, Gamma-aminobutyric acid (GABA) is a neurotransmitter in regulating sleep. Available evidence showed that average brain GABA levels were nearly 30% lower in insomnia patients compared with the normal control [72]. APT improves insomnia by increasing GABA levels [73]. In addition, melatonin is a hormone that plays an important role in maintaining normal sleep. Research found a significant decrease of nocturnal melatonin secretion in insomnia patients [74]. APT may treat insomnia by normalizing the nocturnal secretion of melatonin [75].

### 4.3. Comparison with Other Studies

To date, there has been no synthesis of studies evaluating the favourable benefits of APT for comorbid insomnia. To better understand its benefits, our results were compared with systematic reviews or meta-analyses on APT for primary insomnia. Five previous meta-analyses of the efficacy of APT on primary insomnia [44–48] were examined. Among the five studies, our results for global score on PSQI were consistent with the results identified in two studies [45, 48]. In addition, consistent with two studies [45, 46], the results showed better effective rate after APT compared with control conditions.

Compared with previous reviews of patients with primary insomnia treated with APT [44–48], our review gave a more detailed description of acupressure technique and acupoint selection. The present review demonstrated that the included studies employed various acupressure techniques. The number of main acupoints was also different across studies. It was possible due to insomnia patients with additional disorders. To date, the impact of acupressure technique or the number of main acupoints on comorbid insomnia remains unclear. Further studies are needed to examine the effect of acupressure technique or the number of main acupoints.

### 4.4. Strengths and Limitations

This review has several strengths. The current meta-analysis is the first comprehensive review of the effectiveness of APT on comorbid insomnia given that we systematically searched fifteen databases up to July 2018. Moreover, the included studies were confined to RCTs, which greatly reduced selection bias. Besides, APT was the only intervention selected. Thus, it is easier to determine its positive outcomes compared with multicomponent interventions. In addition, our meta-analysis was rigorously designed and reported following the PRISMA guidelines. To draw a firm conclusion, comprehensive further analyses including subgroup analyses, meta-regression, sensitivity analyses, and publication bias assessment were conducted.

These strengths notwithstanding, several potential limitations also should be noted. First, small sample size, poor study quality of the included studies, and unsatisfactory quality of evidence for the outcomes are the major shortcomings, limiting the power of the study. Therefore, any conclusions from this study should be interpreted with caution. For example, a total of 4,817 articles were identified. Only fourteen were considered for analysis. Furthermore, the included studies had small sample sizes of 40 to 155 participants. In addition, few studies reported their methods of random sequence generation and allocation concealment. Only one study described blinding the personnel and participants, thus both performance and response bias cannot be entirely ruled out. Furthermore, all studies failed to provide trial registration or a protocol, thus selective outcome reporting was thought to having an uncertain risk of bias. According to the GRADE approach, the quality of evidence was low for global score on PSQI, moderate for effective rate, and very low for adverse events. Second, although we conducted subgroup analyses, the heterogeneity could not be thoroughly eliminated. The wide range of comorbidities, different diagnostic criteria used for insomnia, and various treatment regimens may contribute to some of the heterogeneity. To further identify the sources of heterogeneity, more accurate grouping on different confounding factors is needed. Third, our findings were restricted to insomnia patients with medical disorders due to a paucity of information for insomnia patients with psychiatric disorders, which might make generalization of results difficult. The majority of the previous studies on insomnia with psychiatric disorders were excluded because the type of reviews and type of interventions did not satisfy the eligibility criteria. Finally, although data were generally well reported for efficacy of APT in the eligible studies, limited information was reported on adverse events. Moreover, distant effectiveness of APT remained unclear because none of the studies evaluated long-term follow-up outcomes.

### 4.5. Implications for Clinical Practice

Insomnia is one of the most prevalent sleep disorders, especially comorbid insomnia, with potentially harmful consequences [76, 77]. At present, APT has been gaining widespread popularity to improve insomnia symptoms in clinical practice. Our findings indicate that patients with comorbid insomnia may benefit from APT. Nevertheless, given that the evidence in this review was derived from several low quality RCTs, the results are still not definitive. Thus, no firm recommendations can be given for APT in clinical practice. Our study reveals the lack of standardized treatment protocols. Consequently, timing, frequency, and duration of APT to be used are also worth of concern in future research.

### 4.6. Implications for Future Research

Future research designed to further ascertain the efficacy of APT for comorbid insomnia is needed. We carefully assess the included studies and provide the following recommendations:

(1) Future studies should be adherence to the Consolidated Standards of Reporting Trials (CONSORT) [78] guidelines and the Standards for Reporting Interventions in Controlled Trials of Acupuncture (STRICTA) [79] for reporting, which can improve the quality of the publications.

(2) Future studies should also evaluate the quality of evidence and grade the strength of recommendations using the GRADE approach [80].

(3) High-quality RCTs with large sample size are warranted. Furthermore, follow-up assessments should be tested to determine its long-term advantages.

(4) Future studies are necessary to demonstrate clinical effectiveness of APT as monotherapy for insomnia patients with psychiatric disorders.

(5) Randomization, allocation concealment, and blinding should be clearly described to reduce risk of bias.

(6) Standardization of treatment protocols should be conducted in future RCTs to ensure further accumulation of evidence regarding efficacy of APT.

## 5. Conclusions

Taken together, this is the first systematic review and meta-analysis that indicates that APT may be an effective and safe option for comorbid insomnia. However, the paucity of included studies coupled with small sample size, and unsatisfactory quality of methodology and evidence prevent us from drawing a definitive conclusion. Further high-quality and large RCTs with follow-up duration that aim to better delineate the efficacy and safety of APT on comorbid insomnia are required.

## Figures and Tables

**Table 1:** The characteristics of the included studies.

No.	Author (year)	Mean age, y (range)/% female	Duration of insomnia	Design	Sample size (APT/control)	Control intervention	Results reported
1	Huang et al. (2015)	58(NR)/49%	Average 1.875 months	2-parallel arms (MP; sham APT)	90(45/45)	sham APT	APT significantly>sham APT
2	Huo et al. (2014)	53.91(25-82)/28%	NR	2-parallel arms (SV; Estazolam)	64(32/32)	Estazolam 1 mg/day	APT significantly>estazolam
3	Li et al. (2016)	70.17(60-86)/55%	NR	2-parallel arms (SV; Diazepam)	40(20/20)	Diazepam 2.5 mg/day	APT significantly>diazepam
4	Lin et al. (2018)	38.5(18-45)/20%	NR	2-parallel arms (SV; Estazolam)	50(25/25)	Estazolam 1 mg/day	APT significantly>estazolam
5	Qian et al. (2016)	58.69(NR)/35%	NR	2-parallel arms (SV; Estazolam)	40(20/20)	Estazolam 1 mg/day	APT significantly>estazolam
6	Su et al. (2017)	38(26-60)/52%	NR	2-parallel arms (SV; Estazolam)	60(30/30)	Estazolam 1 mg/day	APT significantly>estazolam
7	Wan et al. (2017)	64(42-75)/49%	NR	2-parallel arms (MP; Estazolam)	65(33/32)	Estazolam 2 mg/day	APT significantly>estazolam
8	Wang et al. (2018)	72.19(66-95)/38%	NR	2-parallel arms (SV; Estazolam)	60(30/30)	Estazolam 1 mg/day	No significant difference between APT and estazolam
9	Wu et al. (2012)	NR(46-92)/42%	NR	2-parallel arms (SV; Diazepam)	60(30/30)	Diazepam 2.5 mg/day	APT significantly>diazepam
10	Xing et al. (2014)	55.1(35-67)/48%	3-26 months	2-parallel arms (SV; Estazolam)	64(32/32)	Estazolam 2 mg/day	APT significantly>estazolam
11	You et al. (2015)	74.75(65-82)/40%	NR	2-parallel arms (SV; No treatment)	60(30/30)	No treatment	APT significantly> no treatment
12	Zhang et al. (2016)	63.573(NR)/50%	Average 44.203 days	3-parallel arms ( SV+ music therapy; SV; Estazolam)	155(52/50/53)	Estazolam 2 mg/day	APT+ music therapy significantly > APT and estazolam; no significant difference between APT and estazolam
13	Zhang et al. (2017)	79.65(NR)/42%	NR	2-parallel arms (SV; Estazolam)	60(30/30)	Estazolam 1 mg/day	APT significantly>estazolam
14	Zhou et al. (2013)	NR(18-75)/NR	NR	2-parallel arms (SV; Estazolam)	60(30/30)	Estazolam 1 mg/day	APT significantly>estazolam

APT: auricular plaster therapy; NR: not reported; MP: magnetic pellets; SV: Semen Vaccariae.

**Table 2:** Summary of APT treatment protocol.

No.	Author (year)	Time of pressing	Frequency of pressing	Duration of pressing	Acupressure technique	Main acupoints
1	Huang et al. (2015)	1-2 min each time	3-5 times a day	27 days	Seeds were pressed to produce sourness, distention, numbness, pain and hot sensation that was tolerable to patients	Shenmen
2	Huo et al. (2014)	1-2 min each time	4-5 times a day	Eight weeks	Seeds were pressed to produce mild hot and pain sensation that was tolerable to patients	Shenmen, Occiput, Neurasthenia Area, Neurasthenia Point, Subcortex, Heart, and Deep Sleep Point
3	Li et al. (2016)	NR	3-5 times a day	21 days	Seeds were pressed to produce redness, distention and hot sensation that was tolerable to patients	Shenmen, Occiput, Subcortex, Brain, and Endocrine
4	Lin et al. (2018)	NR	3-5 times a day	6 days	Seeds were pressed to produce sourness, distention, and hot sensation that was tolerable to patients	Shenmen
5	Qian et al. (2016)	At least 2 min each time	3-5 times a day	Two weeks	Seeds were pressed to produce mild distention and pain sensation that was tolerable to patients	Shenmen, Heart, Subcortex, Sympathetic, and Endocrine
6	Su et al. (2017)	NR	3-5 times a day	10 days	Seeds were pressed to produce sourness, pain and hot sensation that was tolerable to patients	Shenmen, Subcortex, Sympathetic, Heart, and Kidney
7	Wan et al. (2017)	1-2 min each time	3-5 times a day	1 month	Seeds were pressed to produce distention, numbness, pain and hot sensation that was tolerable to patients	Shenmen, Endocrine, Heart, and Sympathetic
8	Wang et al. (2018)	3-5 min each time	NR	NR	NR	Shenmen, Sympathetic, Subcortex, and Heart
9	Wu et al. (2012)	3 min each time	3-5 times a day	Four weeks	Seeds were pressed to produce redness, distention and hot sensation that was tolerable to patients	Shenmen, Subcortex, Brain, and Endocrine
10	Xing et al. (2014)	1-3 min each time	3-5 times a day	14 days	NR	Shenmen, Subcortex, Endocrine, and Sympathetic
11	You et al. (2015)	2 min each time	3 times a day	NR	Seeds were pressed to produce sourness, distention, numbness that was tolerable to patients	Heart, Lung, Kidney, Liver, Spleen, and Shenmen
12	Zhang et al. (2016)	0.5-1 min each time	3 times a day	Four weeks	Seeds were pressed to produce hot sensation that was tolerable to patients	Shenmen, Sympathetic, Brain, Heart, Liver, and Kidney
13	Zhang et al. (2017)	3-5 min each time	NR	NR	NR	Shenmen, Sympathetic, Subcortex, and Heart
14	Zhou et al. (2013)	1-2 min each time	4-5 times a day	1 month	Seeds were pressed to produce sourness, distention, numbness and hot sensation that was tolerable to patients	Shenmen, Heart, Sympathetic, and Subcortex

**Table 3:** Quality assessment based on the Jadad scale.

Author (year)	Randomization	Double blinding	Withdrawals and dropouts	Total score
Huang et al. (2015)	1	2	0	3
Huo et al. (2014)	1	0	0	1
Li et al. (2016)	1	0	0	1
Lin et al. (2018)	1	0	1	2
Qian et al. (2016)	1	0	0	1
Su et al. (2017)	1	0	0	1
Wan et al. (2017)	2	0	1	3
Wang et al. (2018)	0	0	1	1
Wu et al. (2012)	1	0	0	1
Xing et al. (2014)	2	0	0	2
You et al. (2015)	1	0	0	1
Zhang et al. (2016)	1	0	1	2
Zhang et al. (2017)	1	0	0	1
Zhou et al. (2013)	1	0	0	1

**Table:** (a) By interchanging random-effect and fixed-effect models

	Standardized Mean Difference (95%CI)	*P-*value	*I* ^*2*^ *-*value, %

Random-effect	-1.13 [-1.48, -0.78]	*P*<0.00001	73%
Fixed-effect	-1.02 [-1.20, -0.84]	*P*<0.00001	73%

**Table:** (b) By omitting one study at a time

Study omitted	Standardized Mean Difference (95%CI)	*P-*value	*I* ^*2*^-value, %

Huo et al. (2014)	-1.17 [-1.57, -0.77]	*P<*0.00001	76%
Li et al. (2016)	-1.10 [-1.48, -0.73]	*P*<0.00001	75%
Lin et al. (2018)	-1.07 [-1.43, -0.71]	*P*<0.00001	73%
Su et al. (2017)	-1.09 [-1.46, -0.71]	*P*<0.00001	74%
Wan et al. (2017)	-1.16 [-1.56, -0.76]	*P*<0.00001	76%
Xing et al. (2014)	-1.11 [-1.50, -0.72]	*P*<0.00001	75%
You et al. (2015)	-1.09 [-1.46, -0.72]	*P*<0.00001	74%
Zhang et al. (2016)	-1.25 [-1.46, -1.03]	*P*<0.00001	12%
Zhou et al. (2013)	-1.17 [-1.56, -0.77]	*P*<0.00001	76%
Combined	-1.13 [-1.48, -0.78]	*P*<0.00001	73%

**Table:** (a) By interchanging random-effect and fixed-effect models

	Risk Ratio (95%CI)	*P-*value	*I* ^*2*^ *-*value, %

Random-effect	1.24 [1.13, 1.36]	*P*<0.00001	37%
Fixed-effect	1.27 [1.18, 1.37]	*P*<0.00001	37%

**Table:** (b) By omitting one study at a time

Study omitted	Risk Ratio (95%CI)	*P-*value	*I* ^*2*^ *-*value, %

Huang et al. (2015)	1.22 [1.11, 1.34]	*P*<0.0001	35%
Huo et al. (2014)	1.23 [1.12, 1.36]	*P*<0.0001	41%
Li et al. (2016)	1.25 [1.13, 1.38]	*P*<0.0001	41%
Lin et al. (2018)	1.23 [1.11, 1.35]	*P*<0.0001	39%
Qian et al. (2016)	1.24 [1.12, 1.37]	*P*<0.0001	42%
Wan et al. (2017)	1.25 [1.13, 1.38]	*P*<0.0001	43%
Wang et al. (2018)	1.26 [1.14, 1.39]	*P*<0.00001	32%
Wu et al. (2012)	1.21 [1.11, 1.33]	*P*<0.0001	31%
Xing et al. (2014)	1.26 [1.13, 1.39]	*P*<0.0001	40%
You et al. (2015)	1.21 [1.11, 1.31]	*P*<0.00001	17%
Zhang et al. (2016)	1.26 [1.15, 1.39]	*P*<0.00001	30%
Zhang et al. (2017)	1.23 [1.12, 1.36]	*P*<0.0001	40%
Zhou et al. (2013)	1.24 [1.12, 1.38]	*P*<0.0001	43%
Combined	1.24 [1.13, 1.36]	*P*<0.00001	37%

**Table 6:** GRADE evidence profile.

**Certainty assessment**							**No. of patients**		**Effect**		**Certainty**
**No. of studies**	**Study design**	**Risk of bias**	**Inconsistency**	**Indirectness**	**Imprecision**	**Other considerations**	**APT**	**Control **	**Relative** **(95**%** CI)**	**Absolute** **(95**%** CI)**	
**Global score on PSQI**											
9	Randomized trials	Serious^a^	Serious^b^	Not serious	Not serious	None	282	284	-	SMD **1.13 lower** (1.48 lower to 0.78 lower)	*⨁⨁*◯◯ LOW
**Effective rate**											
13	Randomized trials	Serious^a^	Not serious	Not serious	Not serious	None	350/407 (86.0%)	277/409 (67.7%)	**RR 1.24** (1.13 to 1.36)	**163 more per 1,000** (from 88 more to 244 more)	*⨁⨁⨁*◯ MODERATE
**Adverse events**											
3	Randomized trials	Serious^a^	Serious^b^	Not serious	Serious^c^	None	3/110 (2.7%)	22/113 (19.5%)	**RR 0.19** (0.01 to 2.89)	**158 fewer per 1,000** (from 193 fewer to 368 more)	*⨁*◯◯◯ VERY LOW

GRADE: Grading of Recommendations Assessment, Development and Evaluation; APT: auricular plaster therapy; PSQI: Pittsburgh Sleep Quality Index; CI: confidence interval; SMD: standardized mean difference; RR: risk ratio.

GRADE Working Group grades of evidence: high quality: further research is very unlikely to change our confidence in the estimate of effect; moderate quality: further research is likely to have an important impact on our confidence in the estimate of effect and may change the estimate; low quality: further research is very likely to have an important impact on our confidence in the estimate of effect and is likely to change the estimate; very low quality: we are very uncertain about the estimate.

^a^Most information is from studies at significant risk of bias (Figure 2). Potential limitations are likely to lower confidence in the estimate of effect.

^b^Substantial heterogeneity in results remains unexplained.

^c^95% CI is wide enough that they overlaps no effect (i.e., 95% CI includes RR of 1.0).
